# Advancements in IR spectroscopic approaches for the determination of fungal derived contaminations in food crops

**DOI:** 10.1007/s00216-014-8145-5

**Published:** 2014-09-26

**Authors:** David McMullin, Boris Mizaikoff, Rudolf Krska

**Affiliations:** 1 Center for Analytical Chemistry, Department for Agrobiotechnology, University of Natural Resources and Applied Life Sciences Vienna, Konrad-Lorenz-Straße 20, 3430 Tulln, Austria; 2 Institute of Analytical and Bioanalytical Chemistry, University of Ulm, Albert-Einstein-Allee 11, 89075 Ulm, Germany

**Keywords:** IR spectroscopy, Mycotoxin contamination, Chemometrics, Rapid methods, Food chain safety

## Abstract

Infrared spectroscopy is a rapid, nondestructive analytical technique that can be applied to the authentication and characterization of food samples in high throughput. In particular, near infrared spectroscopy is commonly utilized in the food quality control industry to monitor the physical attributes of numerous cereal grains for protein, carbohydrate, and lipid content. IR-based methods require little sample preparation, labor, or technical competence if multivariate data mining techniques are implemented; however, they do require extensive calibration. Economically important crops are infected by fungi that can severely reduce crop yields and quality and, in addition, produce mycotoxins. Owing to the health risks associated with mycotoxins in the food chain, regulatory limits have been set by both national and international institutions for specific mycotoxins and mycotoxin classes. This article discusses the progress and potential of IR-based methods as an alternative to existing chemical methods for the determination of fungal contamination in crops, as well as emerging spectroscopic methods.

## Introduction

Mycotoxins are widespread, unavoidable natural contaminants of numerous agricultural food and feed commodities produced by filamentous fungi. Mycotoxins enter the food chain by infecting crops that are directly consumed by humans or indirectly as feed ingredients ingested by animals. Fungal contamination may occur pre-harvest in the field or post-harvest during storage, and may also be influenced by environmental conditions such as humidity and temperature. These contaminations result in very rapid commodity losses and reductions in quality of economically important commodities such as cereal grains and groundnuts. Trichothecenes, fumonisins, and zearalenone produced by *Fusarium* species, aflatoxins produced by *Aspergillus* species, and ochratoxins from *Aspergillus* and *Penicillum* species receive the most attention because of their frequent occurrence and deleterious health effects. These toxins are remarkably stable and are not readily degraded or removed by food processing. Mycotoxins elicit a wide range of toxic activities that adversely affect the health of both humans and animals [1, 2].

Owing to the human health implications posed by mycotoxins, national and international organizations have adopted regulatory limits for individual mycotoxins and mycotoxin classes that influence international trade. The economic burden of crops contaminated with mycotoxins is additionally increased because of regulatory compliance [3, 4]. Legal requirements to adhere to regulatory limits in agricultural commodities have prompted the development of numerous analytical methods for the determination of mycotoxins. The detection of regulated mycotoxins above the specified legal limit can initiate a series of actions that are costly for industry and, subsequently, the consumer [5]. The analytical requirements at different points of food production chains will vary considerably. A regulator may require more comprehensive instrumentation whereas an easy to use rapid diagnostic test may be appropriate for a primary producer. These aspects as well as what defines a rapid test in an industrial setting are discussed in more detail by Miller et al. [5].

Established analytical methods utilized for routine mycotoxin determination include enzyme linked immunosorbent assays (ELISA) [6] and LC-MS/MS [7, 8]. Immunochemical methods such as ELISA rely on antibodies that are typically specific for an individual mycotoxin or class, are relatively quick, easily performed, and are commonly used to screen raw materials [9]. Newer ELISA test kits are commercially available for most of the major mycotoxins at reduced cost per analysis. However, ELISA methods are highly matrix-dependent and cross reactivity may result in false positives. Low sensitivity in complex food matrices can also lead to false negatives compared with sophisticated chromatographic methods. Routine reference methods for mycotoxin quantification include HPLC coupled to mass spectrometry, ultraviolet or fluorescence detection or gas chromatography (GC) with electron capture, flame ionization or mass detectors [10]. LC-MS/MS based methods offer high sensitivity and selectivity for a wide range of chemically diverse mycotoxins in a single chromatographic analysis. However, many of these methods require time-consuming extraction and cleanup steps. The use of organic solvents is usually not feasible in a non-laboratory setting for employee health and safety reasons. Furthermore, grain elevators maintain a particularly dry environment to prevent mold growth, making the use of water not ideal [5]. Comparatively expensive laboratory procedures requiring technical competence and longer result turnover times increase the financial burden placed upon producers demonstrating the safety of their commodities. These methods generate precise, accurate mycotoxin content data; however, their applicability in the field is not economically practical.

Consequently, new methods are being developed to determine mycotoxin contamination in agricultural commodities, as current strategies do not provide rapid and representative measurements, are costly, time-consuming, and cannot be applied in the field. These new approaches include aptamers [10], optical devices [11], and novel spectroscopic methods. Ideally, a food commodity sample should be analyzed “as is” with minimal sample preparation to be practical within an industrial setting. A more universal approach capable of determining mycotoxin contamination in a variety of solid or liquid raw materials would be more appealing. This could be achieved by determining alterations of inherent food sample properties, such as protein, carbohydrate or lipid content, and texture. Implementing an on-site analytical method that is capable of rapid and reliable determination of fungal contaminants with high accuracy within the imposed upper regulatory limits still remains a challenge. There is a benefit to industry and the consumer from a public health perspective to keep noncompliant food or feed commodities from entering into the food chain because of the adverse health effects caused by mycotoxins.

## IR spectroscopic methods for food analysis

Infrared (IR) spectroscopic methods are among the most promising strategies for determining mycotoxin contamination in agricultural commodities or processed food products. IR-based methods are rapid and nondestructive techniques that require minimal technical training and sample preparation. Analysis is usually not labor-extensive, and large quantities of chemicals are not required in comparison to existing sophisticated chromatographic techniques usually requiring advanced technical competence. These intrinsic qualities of IR-based methods render them an attractive option for high throughput analysis of foodstuffs on site.

**Fig. 1:**
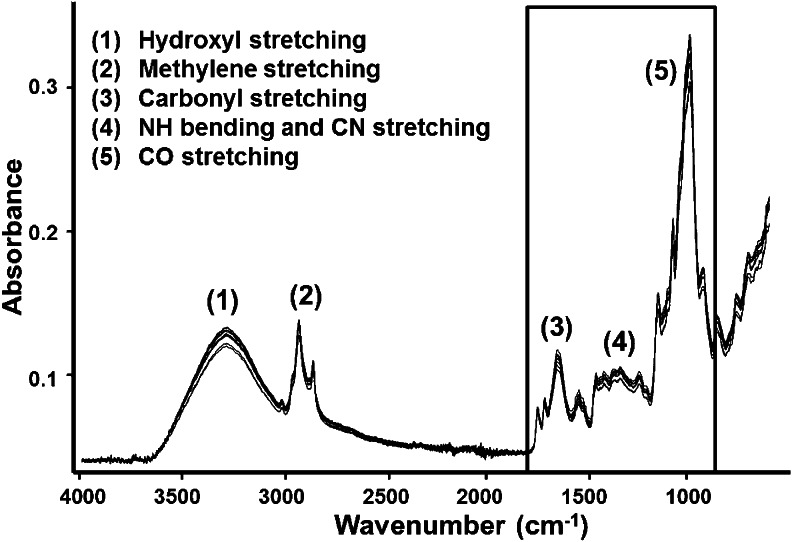
MIR-ATR spectra (*n* = 10) of sieved (<250 μm) maize sample with accompanying band assignments. Within the box, relevant spectral features within the so-called fingerprint region are found

The near infrared (NIR) range, ~13,500 to 4000 cm^–1^, of the electromagnetic spectrum relies on molecular overtones and vibrations of chemical bonds that can be determined by analyzing reflected or transmitted radiation. The signal intensity depends upon radiation absorption and subsequent alterations for example in the hydrogen bond content within in the matrix. The mid infrared (MIR) range, ~4000 to 450 cm^–1^, relies on molecular vibrations of the matrix constituents. Both the NIR and MIR ranges of the electromagnetic spectrum contain selective information for functional groups (e.g., carbonyl, amide, ester, alcohol, methylene, etc.) present within biomacromolecules (e.g., proteins, lipids, carbohydrates, etc.) found in food commodities. Contaminants including mycotoxins currently cannot be directly determined in a complex matrix such as food because of the limited sensitivity of currently available IR-based methods. Fungal contamination in crops can be indirectly assessed by examining the so-called fingerprint region of the MIR spectrum for alterations in intrinsic crop characteristics. Mycotoxigenic *Fusarium* species are known to causes alterations in the carbohydrate and protein content, leading to physical deterioration of the crop attributable to fungal and or mycotoxin accumulation. These physical alterations caused by fungal infection can be investigated by examining differences in the spectral bands; 900–1200 cm^–1^ for carbohydrates and 1200–1750 cm^–1^ for protein amide I and II bands; see Fig. 1 [12–14]. However, these spectral differences need to be correlated to reference measurements such as mycotoxin or ergosterol concentrations.

Interpretation of the MIR fingerprint region of complex food matrices remains difficult for discerning a specific component such as carbohydrates, proteins, and lipids. This necessitates the use of chemometrics to extract and correlate IR spectral information to identify distinct spectral feature changes attributable to fungal contamination. Usually, preprocessing of the spectra is required prior to generation of multivariate chemometric models. Qualitative classification methods include principle component analysis (PCA), cluster analysis, artificial neural networks, and K nearest neighbors. Quantitative multivariate models such as principle component regression/principle component analysis (PCR/PCA) and partial least squares regression (PLS) finally enable comparisons of spectra to independent variables such as mycotoxin concentrations. From reviewing the literature, PLS and PCA are among the most frequently utilized chemometric approaches for authentication of food samples. Chemometric techniques commonly utilized for interpretation and analysis of NIR spectroscopic applications have previously been critically reviewed [15]. Multivariate methods determined to be suitable for fungal contamination in specific matrices could then be packaged into easy-to-use software modules for rapid on-site analysis. However, to generate effective calibration models, reliable IR measurements obtained from large sets of samples with accompanying reference measurements (e.g., LC-MS/MS) indicative of fungal contamination are required. Both the quality of the IR spectra and calibration reference method will greatly influence the chemometric model. Finally, it should be emphasized that proper sampling is the basis for reliable analysis. This is vital for both chemometric model generation and on-site analysis of a commodity, as bulk sampling is the largest source of variance [16].

## Conventional IR spectroscopy for fungal contamination determination

IR, particularly NIR spectroscopy is commonly utilized in the food quality control industry to monitor the physical attributes of numerous cereal grains. Information from the NIR region is used in food quality control to measure protein, oil, and starch content [17]. Many reports classify food samples as either healthy or contaminated, whereas some focus on the use of NIR spectroscopy serving as on-line samplers providing spectral feedback for automated sorting of cereal commodities. Most publications focus on NIR spectroscopy, despite MIR offering a higher content of spectral information. MIR has been applied to the detection of microbial spoilage of meat [18], classification of modified starches [19], apple juice [20], and the determination of fungal contamination in numerous foodstuffs. Pertinent investigations of conventional IR approaches for the determination of fungal contaminations in various food commodities are highlighted here; however, a comprehensive review of the literature on the topic is available [21].

A sample preparation method for increased repeatability of deoxynivalenol (DON) concentration determinations with accompanying IR-attenuated total reflection (ATR) spectroscopy measurements of corn was achieved using the 100–250 μm sieve fraction. Sieved samples were classified correctly 100 % of the time as opposed to 79 % for unsieved samples, identifying this as a possibly necessary step for increasing toxin determination accuracy [22]. IR-ATR spectroscopy has also been applied to determining ochratoxin A (OTA) concentrations in contaminated sultans. A concentration range of 2–50 μg/kg was investigated according to the European Union (EU) regulatory limit of 10 μg/kg. Spectra of sultanas from various origins showed differences in the fingerprint region and in relation to water content. Reproducibility was increased by standardizing the water activity to 0.62. Following PCA analysis, samples of >20 μg/kg could be differentiated from <10 μg/kg or uncontaminated samples [23].

Transmittance NIR spectra of intact wheat kernels followed by PCA indicated that it may be possible to generate calibration models capable of screening for DON at concentrations just above the EU regulatory limit for wheat flour [24]. In a more recent investigation, 262 wheat samples (143 durum and 119 common) derived from 32 different varieties naturally infected by *F. graminearum* or *F. culmorum* were analyzed FT-NIR spectroscopy and HPLC for DON quantification. A qualitative model was developed based on PLS analysis facilitated the differentiation of sound and naturally infected samples when setting the DON threshold at 300 μg/kg. The model was correct 69 % of the time with 65 validation samples; however, it failed with samples around their set DON threshold (16/20). Hence, the utility of FT-NIR to discriminate DON contamination in unprocessed samples below the set EU regulatory level was demonstrated [13].

Automated sorting of intact wheat kernels was achieved by an NIR spectroscopic method that could differentiate samples at a 60 mg/kg DON threshold, which was correct 96 % of the time [25]. Similar sorting of corn kernels in a high throughput bimodal fashion has also been demonstrated. Samples were separated into high (>100 μg/kg) and low (<100 μg/kg) aflatoxin groups with over 95 % efficacy [26]. Evaluation of NIR reflectance bands at 715 and 965 nm could properly identify 98 % of uninfected and 96 % of corn kernels infected by a variety of fungi [27]. These high throughput sorting methods are only applicable in an industrial setting if they are able to discriminate samples near the set regulatory limit for specific mycotoxins within a given commodity.

NIR accompanied by multivariate statistical analysis is capable of predicting the amount of total fungal infection in maize kernels, as well as the ergosterol and fumonisin B1 concentrations in maize meal [28]. In a more recent investigation, 143 Italian corn meal samples were used for chemometric calibration and external validation was performed with 25 unknown samples. The PLS-based chemometric model developed was able to identify samples with >4 mg/kg total fumonisin B1 and B2. This demonstrated the suitability of FT-NIR to discriminate contaminated from safe corn meal [29]. NIR spectroscopy was also used to classify maize samples as positive (>20 μg/kg) or negative (<20 μg/kg) for aflatoxin B1 [30]. Both starch content and moisture were shown to greatly affect NIR measurements when detecting aflatoxigenic fungi on rice leading to a low prediction coefficient, r^2^ = 0.68 [31]. Aflatoxin content was estimated in red chili powder by FT-NIR spectroscopy in diffuse reflectance mode combined with PLS regression analysis. Although high prediction accuracy was achieved between 15 and 500 μg/kg, it should be noted that these samples were spiked and not naturally infected or inoculated [32]. FT-IR spectroscopy has also been applied to the direct measurement of fungal natural products [33] and the identification of filamentous fungi [34].

Spectroscopic methods have received increased attention as an economically viable analytical tool for classifying or estimating both mycotoxin and fungal contamination in agricultural commodities. Most reports focus on classifying samples as safe or contaminated based upon alterations in the spectra determined by multivariate analysis; however, better estimations of mycotoxin concentrations appear promising. Current NIR and MIR methods are limited by reference measurements, extensive calibration for individual food matrices, and complex chemometric data interpretation. The sensitivity of NIR and MIR techniques are still not adequate for reliable mycotoxin estimation at the regulatory limits set by the EU. The development of more robust calibration models with larger data sets that reduce the variability of measurements and advanced chemometric software will contribute to NIR and MIR spectroscopy becoming a more viable technique for fungal and mycotoxin contamination determinations within agricultural commodities.

## NIR hyperspectral imaging

The inherent limitations of reference measurements for food and feed safety as well as quality control have necessitated the development of new analytical technologies. Robust and flexible spectroscopic methods that are amenable to on-site applications in the field or production chain suited for screening of raw materials and final products increasing throughput are required. For food quality and safety, NIR hyperspectral imaging (HSI) is an emerging tool that enables the characterization of complex matrices. HSI, also known as chemical or spectroscopic imaging, integrates both conventional imaging and spectroscopy to generate spatial and spectral information of an object [35]. NIR HSIs are three-dimensional arrays of (*m* x *k* x λ) where *m* and *k* are spatial axes and λ represents the spectral information [36]. HSIs are comprised of hundreds of continuous wavebands for each spatial position of the sample analyzed and its corresponding spectrum [37]. This allows the visualization of biochemical constituents of a particular part of a sample, as regions with similar spectral properties will have similar chemical compositions. A typical HSI system is comprised of an objective lens, spectrograph, camera, acquisition system, illuminator, and computer. Usually, a section of the visible-NIR range (400–1000 nm) is selected and the sample is diffusely illuminated with a tungsten-halogen lamp or LED source. Light is reflected off the sample and separated into its component wavelengths by diffraction optics to generate a two-dimensional image in the spectrograph. The sample is moved past the objective lens on a motorized platform where this process is continuously repeated. Adjacent two-dimensional images acquired are stacked to form a three-dimensional HSIs that can be subsequently processed and analyzed by multivariate statistics [35].

Preprocessing of HSIs is performed to remove nonchemical biases from the relevant information prior to chemometric evaluation. This can remove light scattering effects attributable to inhomogeneities at the sample surface. Chemometrics uses mathematics and statistics to select relevant chemical information by analyzing chemical data, generating useful knowledge of a complex chemical system [38]. Developments in chemometric techniques have facilitated the use of spectroscopic methods because of data reductions allowing more rapid analysis, data extraction, manipulations, and interpretations made possible by increased computing capabilities. This is of consequence for NIR HSI as each sample may constitute thousands of spectra generating very large data files, >50 MB depending on image and spectral resolution, as opposed to the usual average spectra for classic spectroscopic measurements [39]. Chemometric tools used for data classification include PCA, PLS, linear discernment analysis, artificial neural networks, and multi-linear regression. PLS classification has been demonstrated to be the most popular for discriminating HSI data; however, the conventional methods mentioned herein may not always be suitable for HSI data, since they were designed to analyze single spectra. This necessitates the development of specific chemometric strategies to extract spatial information and relate it to physical or chemical characteristics. There is merit in combining NIR HSI with new chemometric tools to increase food chain safety [39].

NIR HSI is currently being implemented in automated food inspection [40], detection of animal proteins in feed [41], characterization of cereals [42, 43], identifying impurities in cereals [41], post-harvest analysis of apples [44], and fungi on food commodities [45, 46]. NIR HSI is quickly maturing into a viable nondestructive method for characterizing fungal contamination on economically important cereals. Visible-NIR HSI was utilized to rapidly discriminate between uninfected and inoculated maize kernels using a 400–1000 nm spectral window. Not only could maize kernels be differentiated as uninfected or *A. niger* and *A. flavus* contaminated, but samples could be separated 48 h post-inoculation. This suggests that early detection of mycotoxigenic fungal contamination in maize is possible [47]. Using 10 different LED lights with emission wavelengths between 720 and 940 nm, ground maize samples were imaged. Using a feed-forward neural network, a significant correlation was observed between fumonisin concentrations and reference measurements [48]. Indirect detection of the fumonisin producer *F. verticilliodes* in maize kernels was achieved with up to 86 % accuracy utilizing NIR HSI [46]. HSI of uncontaminated and *F. verticilliodes* infected maize kernels were analyzed between 1000 and 2498 nm to monitor fungal development within the kernels. Measurements were performed at predetermined intervals and PCA analysis was utilized to determine the degree of infection. Sources of spectral variation caused by infection were observed at 1900 and 2136 nm, indicative of alterations in starch and protein content [42]. The same group additionally used NIR HSI to differentiate three different *Fusarium* species, *F. subglutinans*, *F. proliferatum*, and *F. verticilliodes*, with multivariate image analysis. Changes in the mycelium could be observed as a function of time for each strain and species, which enabled differentiation based on scattering variation that was suspected to be due to protein production differences [49].

Similar experiments have also been performed with wheat. The early detection of FHB was investigated in winter wheat using a 400–1000 nm spectral window followed by PCA. Using HSI, the degree of disease was correctly classified 87 % of the time using the spectral differences in two spectral ranges, 665–675 and 550–560 nm, under laboratory conditions [50]. Eight hundred kernels of Canadian Western Red Spring wheat were segregated as sound, mildly damaged, and severely damaged prior to HSI in the same visible-NIR range. PCA and linear discriminant analysis models were developed to measure the extent of *Fusarium* damage. Wheat samples were categorized up to 92 % of the time correctly using six wavelengths: 484, 567, 684, 817, 900, and 950 nm. The model was correct 84 % in a validation set [51].

NIR HSI is an emerging tool that enables the characterization of complex heterogeneous samples, including food and feed. Many studies utilizing this technology have indicated that early detection of fungal contamination before disease symptoms become evident is possible. Owing to the continued emphasis for the development of accurate, rapid, nondestructive analytical technologies, it is very likely that these techniques will increasingly be adopted for food safety and quality assurance.

## Outlook

**Fig. 2:**
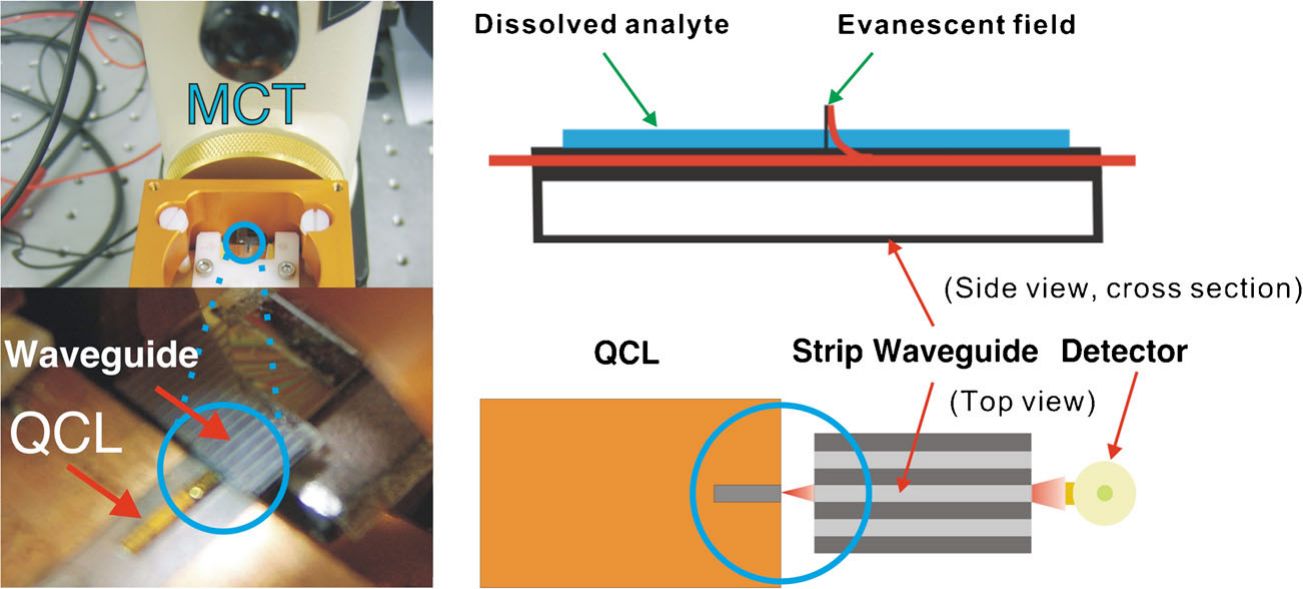
Experimental IR sensor set up coupling a QCL with a GaAs/AlGaAs strip waveguide chip (liquid N_2_-cooled mercury cadmium telluride semiconductor detector)

**Fig. 3:**
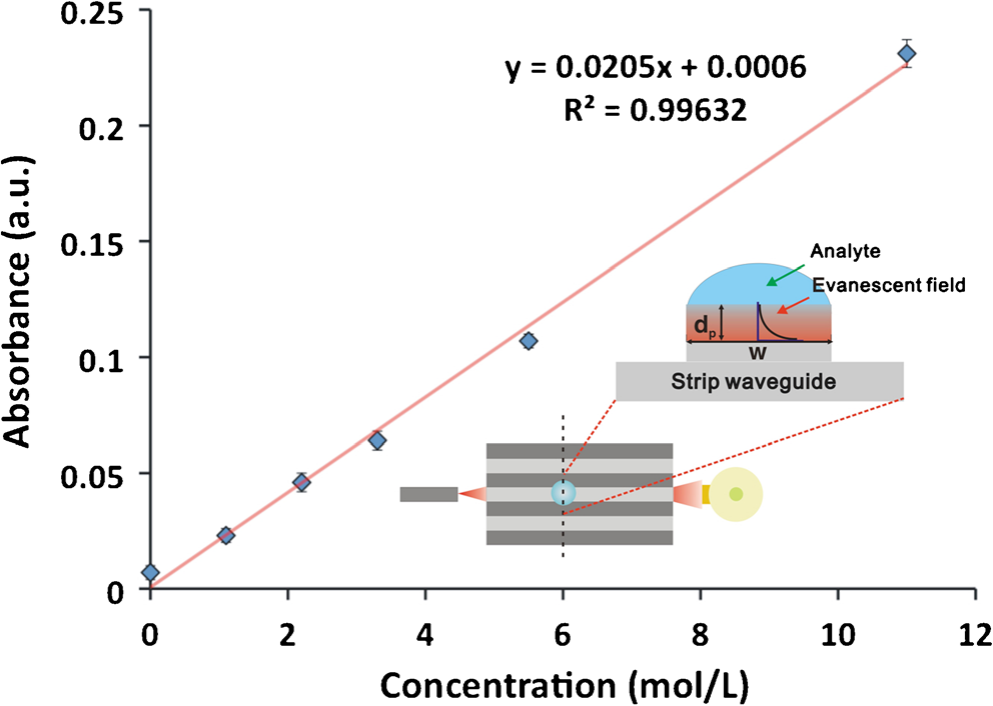
Sensor response to solutions of acetic anhydride in diethylene glycol mono ether. Each 2 nL droplet covers an approximate diameter of 0.44 mm at the strip waveguide surface

The MIR spectral region at 3–20 μm provides fundamental vibrational and rotational fingerprint absorptions of organic molecules offering inherent molecular selectivity. This renders the spectral window attractive for optical sensing applications including the analysis of complex biological matrices such as food or feed [52]. An MIR sensing system utilizing waveguide-based optical sampling principles is typically comprised of a radiation source, a waveguide to propagate radiation that frequently serves simultaneously as a transducer and a detector. A major limitation of conventional MIR measurements is the achievable sensitivity because of the low intensity of conventional light sources. Advancements in MIR laser technology and, in particular, quantum cascade laser (QCL) technology have rendered them the most promising radiation source for advanced IR sensing applications. Figure 2 depicts an experimental IR set-up encompassing a QCL, planar waveguide, and detector. QCLs offer compact dimensions, high output power, long lifetime, broad tunability (>200 cm^–1^ for a single laser), room temperature operation, and access to almost the entire MIR spectral band [52, 53]. QCLs are semiconductor lasers that generate light emission via intersubband transitions of electrons within the conduction band, taking advantage of a series of quantum heterostructures [54]. In the case of a waveguide-based optical sensor, the transducer is a vital component that translates the chemical or biological signal into an optical signal that may be further processed. Here, it is the waveguide–analyte interface where photons interact with molecular sample constituents that determines the reproducibility of the measurements and quality of the analytical signal. Most MIR sensors are based on internal reflection spectroscopy or, more specifically, ATR techniques. ATR spectroscopy in the MIR range is well established and conventionally uses crystalline materials such as zinc selenide, germanium, or silicon. These configurations allow for a defined yet frequently limited number of internal reflections (i.e., interactions) with the sample present at the waveguide surface. Total internal reflection occurs if light at an angle of incidence exceeding the critical angle is reflected at the interface of an optically denser waveguide and an optically thinner surrounding medium (e.g., an analyte matrix present in liquid or solid state at the waveguide surface). Owing to the optical conditions, electromagnetic radiation propagating along the waveguide leaks into the adjacent environment, which is called an evanescent field. The evanescent field exponentially decays into the sample adjacent to the waveguide with a penetration depth largely determined by the wavelength of the radiation (i.e., few micrometers in the MIR regime) [55]. Until recently, waveguide technology in the MIR band was significantly less developed compared with the visible and NIR spectral regions. A new generation of semiconductor GaAs/AlGaAs thin-film IR waveguides grown by metal–organic vapor-phase epitaxy (MOPVD) have been developed that are transparent throughout most of the MIR region [56, 57]. In addition, novel planar waveguides comprised of mercury-cadmium-telluride (MCT) also transparent through the MIR region have been reported [53]. Both ideally combine with QCL light sources and may be tailored, frequency-matched to the characteristics of the light source, thereby maximizing the photon–sample interaction. Conventional waveguides are typically much thicker than the applied light wavelength meaning only a few total reflections occur within the device, thereby limiting sensitivity. To improve the signal-to-noise ratio, a thin-film frequency-matched waveguide is required to ensure that most of the energy is transported within the evanescent field where, upon interaction with the analyte, the analytical signal is actually generated (i.e., via evanescent field absorption spectroscopy). This has been demonstrated with the newly developed GaAs/AlGaAs as well as the MCT thin-film waveguides, both providing single-mode behavior and illustrated in Fig. 3. Increasing the intensity of the evanescent field directly affects the achievable signal-to-noise ratio, thereby providing potentially more sensitive measurements in the MIR range compared with conventional (i.e., macroscopic) IR-ATR spectroscopies. Coupling QCLs with these novel thin-film waveguides provides an intriguing opportunity to generate higher quality IR data, which is of particular interest for in-field usage of MIR sensing systems, given the potential of miniaturization by combining thin-film MIR waveguide technology with QCLs. Consequently, it appears that the on-site characterization and analysis capabilities for food or feed samples in a high throughput manner are on the horizon.

Advances in microfabrication and miniaturization of sensor components such as tunable QCLs, detectors and integrated optics, including novel thin-film waveguides, will aid in advancing the applicability of such techniques, particularly in the environment of food and feed analysis. Whereas conventional spectroscopic methods are frequently confined to laboratory settings, combining QCLs with frequency-matched thin-film MIR waveguides has the potential to offer highly sensitive, selective spectral information that can be packaged into miniaturized sensing devices. A currently ongoing research project funded by the European Union (MYCOSPEC; mycospec.eu) is aiming at maturing these technologies toward in-field deployable rapid detection systems. These features would make MIR sensing devices attractive options for the rapid, on-site, determination of fungal contamination in both solid and liquid foodstuffs.
